# International non-governmental organizations’ provision of community-based tuberculosis care for hard-to-reach populations in Myanmar, 2013–2014

**DOI:** 10.1186/s40249-017-0285-3

**Published:** 2017-03-24

**Authors:** Kyaw Thu Soe, Saw Saw, Johan van Griensven, Shuisen Zhou, Le Win, Palanivel Chinnakali, Safieh Shah, Myo Myo Mon, Si Thu Aung

**Affiliations:** 1 Department of Medical Research, (Pyin Oo Lwin Branch), Ward 16, Near Anisakhan Airport, Pyin Oo Lwin Township, Mandalay Region Myanmar; 2 Institute of Tropical Medicine, Nationalestraat 155, Antwerp, 2000 Belgium; 3 National Institute of Parasitic Diseases, Chinese Center for Disease Control and Prevention, 207 Ruijin Er Road, Shanghai, 200025 People’s Republic of China; 4 Jawaharlal Institute of Postgraduate Medical Education and Research, No-1, Vinayagar kovil Street, Kathirkamam, Puducherry 605009, India; 5 Operational Research Unit, Médecins Sans Frontières, Operational Research Unit, 68, rue de Gasperich, Luxembourg City, L-1617 Luxembourg; 6 National Tuberculosis Programme, Disease Control Unit, Nay Pyi Taw Bed 1000 Hospital, Zabu Kyatthayay road, Zabu Thiri Township, Nay Pyi Taw, Myanmar

**Keywords:** Operational research, Community, Tuberculosis, Volunteers, Contribution, International non-governmental organizations, Myanmar

## Abstract

**Background:**

National tuberculosis (TB) programs increasingly engage with international non-governmental organizations (INGOs), especially to provide TB care in complex settings where community involvement might be required. In Myanmar, however, there is limited data on how such INGO community-based programs are organized and how effective they are. In this study, we describe four INGO strategies for providing community-based TB care to hard-to-reach populations in Myanmar, and assess their contribution to TB case detection.

**Methods:**

We conducted a descriptive study using program data from four INGOs and the National TB Program (NTP) in 2013–2014. For each INGO, we extracted information on its approach and key activities, the number of presumptive TB cases referred and undergoing TB testing, and the number of patients diagnosed with TB and their treatment outcomes. The contribution of INGOs to TB diagnosis in their selected townships was calculated as the proportion of INGO-diagnosed new TB cases out of the total NTP-diagnosed new TB cases in the same townships.

**Results:**

All four INGOs implemented community-based TB care in challenging contexts, targeting migrants, post-conflict areas, the urban poor, and other vulnerable populations. Two recruited community volunteers via existing community health volunteers or health structures, one via existing community leaderships, and one directly involved TB infected/affected individuals. Two INGOs compensated volunteers via performance-based financing, and two provided financial and in-kind initiatives. All relied on NTP laboratories for diagnosis and TB drugs, but provided direct observation treatment support and treatment follow-up.

A total of 21 995 presumptive TB cases were referred for TB diagnosis, with 7 383 (34%) new TB cases diagnosed and almost all (98%) successfully treated. The four INGOs contributed to the detection of, on average, 36% (7 383/20 663) of the total new TB cases in their respective townships (range: 15–52%).

**Conclusion:**

Community-based TB care supported by INGOs successfully achieved TB case detection in hard-to-reach and vulnerable populations. This is vital to achieving the World Health Organization End TB Strategy targets. Strategies to ensure sustainability of the programs should be explored, including the need for longer-term commitment of INGOs.

**Electronic supplementary material:**

The online version of this article (doi:10.1186/s40249-017-0285-3) contains supplementary material, which is available to authorized users.

## Multilingual abstract

Please see Additional file 1 for translations of the abstract into the five official working languages of the United Nations.

## Background

Tuberculosis (TB) is a communicable disease, which remains a leading cause of mortality. In 2013, there were nine million cases of TB and 1.5 million TB-related deaths globally, the vast majority of which occurred in low- and middle-income countries. Approximately three million people develop TB every year in the World Health Organization (WHO) Southeast Asia Region [1]. Myanmar, a country in Southeast Asia, is listed as one of 30 countries with a high burden of TB, TB/HIV, and multi-drug resistant TB [1].

In 2006, the WHO launched the STOP TB Strategy consisting of six components to achieve a wide expansion of high-quality directly observed treatment, short-course (DOTS). One of the strategies is to “Empower people with TB, and communities through partnership” [2]. The strategy aims to increase advocacy, communication, and social mobilization; increase involvement of communities and patients in TB care and prevention; and promote and enable health-seeking behavior among all people in the country. The importance of community involvement was emphasized again in the End TB Strategy launched in 2015 [3]. Community-based TB care (CBTC), which aims to involve the community in TB prevention and care activities, is a vital part of this strategy, especially when targeting hard-to-reach and/or remote populations.

CBTC refers to TB control services implemented by the National Tuberculosis Control Program (NTP) by collaborating with local and international non-governmental organizations (NGOs) through community health volunteers. The specific objectives of CBTC are four-fold: 1) to improve TB case finding, 2) to improve case holding 3), to increase community awareness about TB, and 4) to empower communities for health through TB care.

Since 2011, the Myanmar NTP has increasingly engaged in partnerships with both international non-governmental organizations (INGOs) and local NGOs to scale up CBTC. Currently, four INGOs are fully involved in CBTC [4]. INGOs can complement NTP activities by specifically focusing on areas where TB care provision is challenging for various reasons such as limited resources (man, money, materials) for TB care, difficult in transportation and poor accessibility to TB diagnosis and management services, and poor knowledge and attitude of Myanmar people on TB.

While international guidelines have been developed for CBTC and program indicators have been defined, there is a dearth of publications from Southeast Asia on what role CBTC plays in national TB programs in general and how INGOs provide this type of care in particular. Detailed information on program activities and performance would be directly relevant for both INGOs and the NTP to identify gaps and define strategies for further improvement. Moreover, INGO activities will ultimately be taken over by national actors, making assessments of the extent and variability in the types of activities and approaches offered by the different INGOs particularly relevant for national programs.

This study was designed to assess INGOs’ provision of CBTC in Myanmar. Using routinely collected program data from 2013–2014 from the four INGOs involved in CBTC, we report on 1) their strategies and activities; 2) the number of cases tested, diagnosed, and successfully treated; and 3) their contribution towards TB case detection in relation to NTP activities.

## Methods

### Study design

This was a cross-sectional descriptive study, which used available routine data of four INGOs and the NTP in Myanmar in 2013–2014.

### Setting

Myanmar, located in Southeast Asia with a total of around 51 million inhabitants, has a high burden of TB [5]. In 2013, the incidence was 373 per 100 000 inhabitants and the prevalence was 473 per 100 000 inhabitants [4].

The country is divided into 15 region/states, 74 districts and 412 townships and sub-townships, 398 towns, 3 065 wards, 13 619 village tracts, and 64 134 villages [5]. Each township has at least one hospital and several rural health centers. The NTP under the Department of Public Health (Ministry of Health and Sports) carries the overall responsibility for TB control in Myanmar. The NTP currently involves 14 regional and state TB centers with 101 TB teams at the district and township levels. All townships in Myanmar have been covered with the DOTS strategy since 2003. The TB control activities are implemented at the township level as part of integrated primary health care provision [4].

**Fig. 1:**
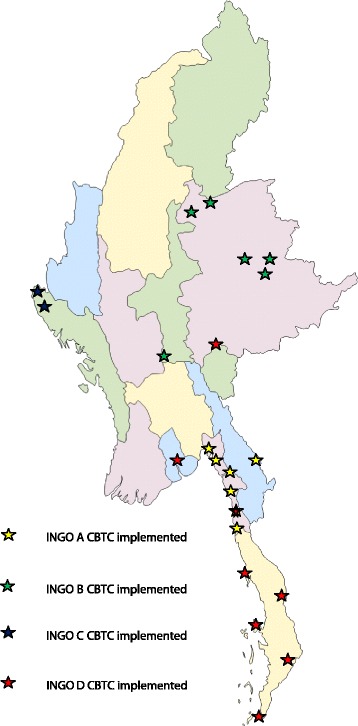
Map showing townships of Myanmar where the four INGOs were implementing CBTC in 2013–2014 (source: http://www.d-maps.com)

Each of the four INGOs involved in CBTC is active in different, non-overlapping implementation areas. These areas are selected jointly by the INGOs and the NTP, preferentially targeting remote and hard-to-reach areas, which are marked by challenging TB care delivery, despite a relatively high TB burden. The four INGOs involved in this study together provided CBTC in 22 townships during 2013–2014. The townships where these four INGOs were implementing CBTC are shown in Fig. 1.

### CBTC provided by the INGOs

The elements of CBTC are community mobilization, recruitment and training of community volunteers, raising awareness on TB within the community through volunteers, detection of presumptive TB cases by volunteers, referral to the NTP centers for TB diagnosis, attending DOTS by the volunteers until completion, counseling of TB patients on treatment adherence, and nutritional and financial support for TB patients.

The NTP has developed guidelines for CBTC with the aim of providing a harmonized approach among the different organizations working on CBTC in Myanmar. The guidelines for CBTC for basic health staffs in Myanmar can be outlined as: rationale for implementing CBTC in Myanmar, the objectives of CBTC, stakeholder’s involvement in CBTC, planning CBTC at township level (microplan), essential steps for CBTC implementation, incentives or enablers for CBTC, supervision, monitoring, and evaluation for CBTC [6].

### Study population

The study population included all presumptive TB cases referred for TB testing by the INGOs or tested by the NTP centers in the same implementing area between 2013 and 2014.

### Data source and collection

Every 12 months, all INGOs send reports with aggregated data on their activities to the NTP and funding agencies, from which the data for this study were sourced. Additionally, and specifically, INGOs provided details of the content and scope of their CBTC activities. For each INGO, the following data were collected: geographical coverage; program objectives, strategies, and activities; number of presumptive TB cases referred for TB diagnosis; presumptive cases tested for and diagnosed with TB; number of TB cases tested for HIV; and number of TB patients receiving DOTS and their treatment outcomes. The total number of presumptive cases tested for TB and the number of TB patients amongst the tested presumptive TB cases registered with the NTP (including the cases reported by INGOs) per township were collected from the annual NTP report.

For TB diagnosis, we report on all new TB cases diagnosed and the number of bacteriologically confirmed cases. A TB outcome was classified as cured, completed, or other (treatment failed, died, lost to follow up, not evaluated). The treatment success rate was defined as the proportion of cases cured or completing TB treatment out of the total TB cases registered for treatment.

### Analysis and statistics

In descriptive analysis, we summarized the number of presumptive TB cases tested, and those diagnosed with TB and their treatment outcomes for each INGO. The proportions of presumptive TB cases tested and TB cases of all four INGOs in their selected townships out of the total cases reported by the NTP in the same townships were calculated using Microsoft Excel.

## Results

The strategies and approaches of the four INGOs involved in CBTC are presented in Table 1. All four targeted hard-to-reach or highly mobile communities. Two mainly targeted migrant workers at border regions, one focused on people living in a conflict zone, and one on the population in an insecure region. Two INGOs targeted suburban areas located next to of rural areas. There were also clear differences in the size of the target population, with the number of targeted townships ranging from two to eight.

**Table 1:** Strategies and activities of INGOs providing CBTC in Myanmar, 2013–2014

	INGO A	INGO B	INGO C	INGO D
Target population	Migrants and migrant-affected communities (rural/suburban)	Hard-to-reach rural communities	Rural communities including conflict areas with high numbers of refugees	Rural and suburban migrant communities
No. of townships	7	6	2	8
Population size	1 434 504	726 519	869 743	1 432 463
Volunteer recruitment	Using existing community structures (village leaders) to recruit outreach health workers who are trained in TB care	Training of existing community health volunteers on TB	Volunteer recruitment via township health departments	Establishment of self-help groups: TB infected or affected people as volunteers
Volunteer support	Salary-like incentives	In-kind and minor financial incentives	Performance-based payments	Performance-based payments
Supervision of volunteers	Township	Township	Township	Township
TB awareness raising	+	+	+	+
TB case detection	+	+	+^a^	+
DOTS provision	+	+	+	+
HIV testing	+	-	-	-
Patient support	+++	++	++	++
Behavioral change activities	Health education via radio, health education sessions in communities	Health education sessions in communities	Health education sessions in communities	Health education sessions in communities

^a^INGO C supports diagnostic facilities of township health departments in addition to provision of direct TB screening through decentralized screening centers and mobile clinics

While the core activities of the INGOs were fairly similar, a clear difference was observed in terms of volunteer recruitment and types of volunteers. INGOs B and C worked via existing community health volunteers or health structures, INGO A recruited outreach workers via existing community structures and leaders, while INGO D organized its activities around people infected with or affected by TB. All referred to the township health hospital for TB diagnosis and treatment, with INGO C additionally supporting decentralized clinics for TB diagnosis. Only one INGO conducted activities promoting HIV testing in TB patients.

**Fig. 2:**
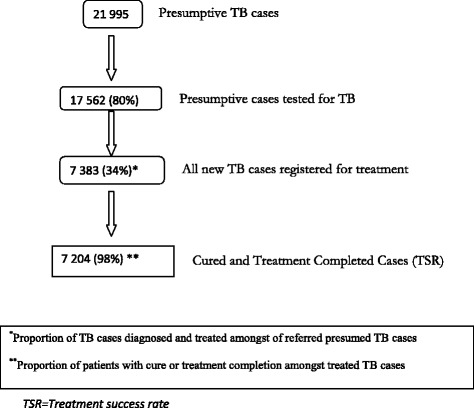
TB diagnosis, type of TB, and treatment outcomes under the CBTC programs of four INGOs in Myanmar, 2013–2104 (covering 22 townships)

Between 2013 and 2014, a total of 21 995 presumptive TB cases were referred by the four INGOs, of which 17 562 (80%) were tested. Out of the referred cases, a total of 7 383 (34%) new TB cases were registered for TB treatment. Of these, almost all (98%) were successfully treated (see Fig. 2). Out of the 7 383 new TB cases, 2 138 (29%) were confirmed bacteriologically. The INGO promoting HIV testing reported HIV infection in 268 (11%) of the 2 505 confirmed TB cases who underwent testing.

**Fig. 3:**
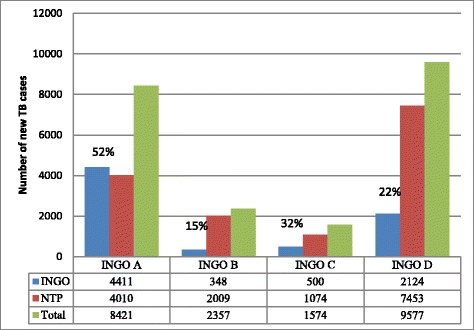
Contribution of INGOs in the detection of new TB cases in Myanmar, 2013–2104. Coverage of townships for the different INGOs: INGO A: 7, INGO B: 6, INGO C: 2, INGO D: 8

The four INGOs combined contributed to the detection of, on average, 36% (7 383/20 663) of the total new TB cases in their respective townships. This ranged from 15% (INGO B) to 52% (INGO A) (see Fig. 3).

Between the stages of referral and testing, it was noted that 20% of presumptive TB cases did not receive any kind of TB testing. The possible reason might be that some TB patients in this study were migrants and they might have gone to other places before receiving a TB test. The nature of migrants is moving one place to another place based on their jobs. Although the people are suspected to have TB, and they are encouraged to take TB test, they might not receive it. Because, these migrant people might move to other place before they go and take the TB testing.

People with presumptive TB symptoms might not have been aware of the importance of TB testing, so they might have refused to be tested.

## Discussion

This study aimed to describe the strategies INGOs involved in CBTC use for hard-to-reach populations and/or in complicated contexts, and their contributions to TB case detection. All INGOs worked with community volunteers, but there were clear differences in how these volunteers were identified and recruited. All INGOs substantially contributed to TB case detection and complemented NTP activities, with good treatment outcomes observed.

The End TB Strategy aims to achieve ambitious increases in TB case detection and reductions in TB-associated deaths. The END TB strategy 2016–2035 vision is “A world free of TB: Zero deaths, disease and suffering due to TB”, the goal is “End the global TB Epidemic”, the milestones for next 10 years (2025) are 75% reduction in TB deaths (compared with 2015), 50% reduction in TB incidence rate (less than 55 TB cases per 100 000 population), and no affected families facing catastrophic costs due to TB.

This strategy stipulates the involvement of all actors involved in TB, while at the same time aims to engage communities in TB control. This might be especially required in areas where ensuring access to TB services is most challenging, or where traditional health facility-based strategies might be insufficient. The relatively high contribution to TB case detection in the respective townships confirms that CBTC activities addressed an unmet need.

Other studies also support the role of CBTC in TB care. In a recent meta-analysis, involvement of community volunteers was associated with improved outcomes compared to standard facility-based TB care [7]. Previous studies conducted in Myanmar also confirmed the role of community involvement in early TB case detection, demonstrating that it not only positively impacts on case detection, but also enhances community awareness about TB [8–12]. A qualitative study from Myanmar showed that organizing self-help groups (as done by INGO D) resulted in effective empowerment of TB patients by providing support, supervision, and appraisal, which can have a long-lasting impact on their role in CBTC [13].

The INGOs in our study worked in diverse settings, targeting different populations. Their approaches and strategies also differed, probably adapted to the specific settings or in line with their specific expertise. While for national TB programs standardization is vital, INGOs are naturally diverse in their approaches, and can also more easily adapt and fine tune their activities to various contexts. As such, NGOs are a key partner for national TB programs, playing a complementary role in TB control in challenging contexts with vulnerable populations.

However, INGO involvement also raises issues of sustainability, related to the planned scaling down of activities or more abrupt interruptions due to funding gaps. As a consequence, it is vital for NTPs to anticipate the handing over of projects from the onset, to local NGOs or to be taken by the NTP.

INGOs also tend to have strategies, procedures, and modus operandi (e.g. in terms of patient support and staff incentives) that are different from those applied by local NGOs or the NTP. Incentives for volunteers would be the major factor contributing to the success of INGOs providing CBTC, although more evidence is required on this point. Constructive discussions between all stakeholders on how INGO strategies can be adapted and integrated into local contexts to ensure sustainability and easy handover would be most useful.

One of the strengths of this study relates to the fact that INGOs were willing to share detailed information on their strategies and program activities. The study also provides information on how INGO-related CBTC can address needs across a range of contexts and target populations.

There are also a number of important limitations to acknowledge. First, it was difficult to validate the data in the INGO reports. We also could not demonstrate the additional yield in TB diagnosis related to the activities of INGOs and could not report indicators of all their activities. We only had information on the rate of detection of new TB cases. Finally, qualitative research on patients’ and volunteers’ perspectives and levels of satisfaction would have enriched the study. Such work is ongoing.

## Conclusion

In conclusion, INGOs involved in CBTC were successful in facilitating TB care in challenging contexts in Myanmar. More work needs to be done to define what is a good balance between standard NTP activities, INGO involvement, and the role and contribution of the community. To ensure sustainability of INGO activities and to ease handovers, open and transparent discussions between INGOs, local NGOs, and national TB programs should be organized from the onset.

## Additional file

Additional file 1: Multilingual abstracts in the five official working languages of the United Nations. (PDF 740 kb)

## Abbreviations

CBTC: Community-based tuberculosis care
DOTS: Directly observed treatment, short-course
HIV: Human immunodeficiency virus
INGO: International non-governmental organization
NGO: Non-governmental organization
NTP: National Tuberculosis Control Program
TB: Tuberculosis
WHO: World Health Organization
