# Supplementary material for: International non-governmental organizations’ provision of community-based tuberculosis care for hard-to-reach populations in Myanmar, 2013–2014
Source: Infect Dis Poverty. 2017 Mar 24;6:69. doi: 10.1186/s40249-017-0285-3 (PMC5364576; doi:10.1186/s40249-017-0285-3)
Supplement: Additional file 1: — Multilingual abstracts in the five official working languages of the United Nations. (PDF 740 kb) [file 40249_2017_285_MOESM1_ESM.pdf]

Translation of the abstract into the six official working languages of the United Nations

توفير المنظمات الدولية غير الحكومية الرعاية المجتمعية لمرضى السل للسكان الذين يصعب الوصول إليهم في ميانمار، 2013-2014

كياو ثو سو، ساو ساو، يوهان فان جرينزفن، شويسن تشو، لي وين، بالانيفل تشيناكالي، صافية شاه، ميو ميو مون، سي ثو أونج

ملخص

خلفية: تشترك برامج السل الوطنية على نحو متزايد مع المنظمات الدولية غير الحكومية، وخاصة لتوفير رعاية لمرضى السل في الأماكن ذات البيئات المعقدة حيث قد تكون هناك حاجة لإشراك المجتمع المحلي. في ميانمار، ومع ذلك، هناك بيانات محدودة عن كيفية إعداد البرامج المجتمعية للمنظمات غير الحكومية الدولية ومدى فاعليتها. في هذه الدراسة، وصفنا أربع استراتيجيات يمكن للمنظمات الدولية غير الحكومية استخدامها لتوفير الرعاية المجتمعية لمرضى السل من السكان الذين يصعب الوصول إليهم في ميانمار، وتقييم مساهمتها في الكشف عن حالات السل.

الأسلوب: أجرينا دراسة وصفية باستخدام بيانات برامج أربعة منظمات غير حكومية دولية والبرنامج الوطني لمرض السل في 2013-2014. لكل منظمة دولية غير حكومية، استخرجنا المعلومات عن نهجها وأنشطتها الرئيسية، وعدد من حالات السل المحتملة المحالة إليها والجاري فحصهم للسل، وعدد من المرضى الذين شخص إصابتهم بمرض السل ونتائج العلاج. تم احتساب مساهمة المنظمات غير الحكومية الدولية في تشخيص السل في البلدات التي تعمل بها من نسبة حالات السل الجديدة التي شخصتها المنظمات الدولية غير الحكومية، من مجموع حالات السل الجديدة التي شخصها البرنامج الوطني لمرض السل في نفس البلدات.

النتائج: جميع المنظمات غير الحكومية الدولية الأربعة التي تقوم رعاية مرضى السل المجتمعية في بيئات صعبة، تستهدف المهاجرين ومناطق ما بعد الصراع، والفقراء في المناطق الحضرية، وغيرهم من الفئات الضعيفة من السكان. اثنتان تستعينان بمتطوعين من المجتمع عن طريق متطوعي صحة المجتمع أو المرافق الصحية الحاليين، إما عن طريق القيادات المجتمعية القائمة، أو عن طريق المصابين/الأفراد المتضررين من السل حاليا. استعاضت اثنتان من المنظمات غير الحكومية الدولية عن المتطوعين بالتمويل القائم على الأداء، واثنتان قدمتا مبادرات مالية وعينية. اعتمدت كل المنظمات على مختبرات البرنامج الوطني لمرض السل لتشخيص السل والمخدرات، لكنها لم تقدم الدعم المباشر لمراقبة العلاج ومتابعة العلاج.

وتمت إحالة ما مجموعه 21995 حالة مشتبه إصابتها بالسل للتشخيص، شُخص 7383 (34%) حالة سل الجديدة وكلها تقريبا (98٪) عولجت بنجاح. ساهمت أربعة منظمات غير حكومية دولية في الكشف في المتوسط عن 36٪ (20663/7383) من مجموع حالات السل الجديدة في بلداتهم (المدى: 15-52٪).

الاستنتاجات: الرعاية المجتمعية لمرضى السل بدعم من المنظمات غير الحكومية الدولية تحقق نجاحا في اكتشاف حالات السل في السكان الذين يصعب الوصول إليهم والمعرضين للإصابة بالمرض. وهذا أمر حيوي لتحقيق أهداف منظمة الصحة العالمية الاستراتيجية للقضاء على السل. ينبغي إيجاد استراتيجيات لضمان استدامة البرامج، بما في ذلك الحاجة إلى الالتزام على المدى الطويل من المنظمات غير الحكومية الدولية.

Translated from English version into Arabic by Mahmoud Sami, through

TRANSLATORS WITHOUT BORDERS

2013-2014 年国际非政府组织为缅甸难以接触到人群提供社区结核病保健的干预效果

Kyaw Thu Soe, Saw Saw, Johan van Griensven, Shuisen Zhou, Lei Win, Palanivel Chinnakali, Safieh Shah,Myo Myo Mon, Si Thu Aung

摘要

引言：国家结核病（TB）项目越来越多地与国际非政府组织（INGOs）合作，尤其是针对有社区参与需求的复杂社区提供 TB 保健。然而，针对缅甸的 INGO 社区项目的组织管理和干预效果的文献报道很少见。因此，本研究描述了在缅甸为难以接触到人群提供社区 TB 保健干预的 4 个 INGO 策略，评估 INGO 对 TB 病例检测的贡献。

方法：本研究采用了 4 个 INGOs 和国家 TB 项目（NTP）的项目数据进行描述性研究。提取每个 INGO 的方法和关键活动的数据，疑似 TB 病例数和接受 TB 检查的人数，以及确诊 TB 的患者数量和治疗结果等信息。通过计算 INGO 诊断的新发 TB 病例数与 NTP 在同一镇区诊断的总新发病例数的比例来评估 INGO 在所选乡镇诊断 TB 的贡献。
结果：4 个 INGO 均在具有挑战性的环境中实施社区 TB 保健，针对包括移民、冲突后地区、城市贫民和其他弱势群体。通过现有的社区保健志愿者和卫生机构招募两名社区志愿者，一名通过现任的社区领导招募，一名为直接涉及 TB 感染/受影响的个人。两个 INGOs 通过绩效融资来补偿志愿者，而另外两个 INGOs 通过现金或实物补偿。4 个 INGOs 均依赖 NTP 实验室进行诊断和提供 TB 药物，但提供直接观察治疗和预后随访。一共有 21 995 例 TB 疑似病例由 4 个 INGOs 确诊为 TB，其中 7 383 例（34%）为新发病例，且几乎所有病例（98%）被成功治愈。各乡镇种所有新发 TB 病例的 36%（7 383/20 663）（范围：15-52%）是由这 4 个 INGOs 检测出来的。
结论：INGOs 提供的社区 TB 保健有效地检测难以到达和弱势人群中的 TB 病例。这对实现 WHO 消除 TB 策略的目标至关重要。INGOs 长期保障的需求等确保项目可持续性的策略应作进一步研究。

Translated from English version into Chinese by Hong Tu, edited by Pin Yang

CHINESE CENTER FOR DISEASE CONTROL AND PREVENTION
NATIONAL INSTITUTE OF PARASITIC DISEASES

Interventions d'organisations non gouvernementales internationales pour le traitement de la tuberculose parmi les populations isolées du Myanmar, 2013-2014

Kyaw Thu Soe, Saw, Johan van Griensven, Shuisen Zhou, Lei Win, Palanivel Chinnakali, Safieh Shah, Myo Mon, Si Thu Aung

Résumé
Contexte: Les programmes nationaux de lutte contre la tuberculose impliquent de plus en plus des organisations non gouvernementales internationales (ONGI), notamment pour dispenser des soins dans des environnements complexes où il peut être nécessaire d'impliquer la population dans le processus. Toutefois, on ne dispose que de peu d'informations sur l'organisation de ces programmes communautaires des ONGI au Myanmar ni sur leur efficacité. Dans cette étude, nous décrivons quatre stratégies communautaires pour dispenser des soins antituberculeux dans des régions isolées du Myanmar et évaluons la contribution de ces ONGI à la détection des cas de tuberculose.
Méthodes: Nous avons mené une étude descriptive à partir des données de programme de quatre ONG internationales et du programme national de lutte contre la tuberculose (PNT) en 2013-2014. Pour chaque ONGI, nous avons extrait les informations relatives à son approche et à ses principales activités, le nombre de cas présumés de tuberculose adressés aux centres de soins et en cours de dépistage et le nombre de patients diagnostiqués positifs ainsi que l'issue de leur traitement. La contribution des ONGI au diagnostic de la tuberculose dans les villages qu'elles avaient choisis a été calculée comme la proportion de nouveaux cas de tuberculose diagnostiqués par les ONGI par rapport au total des nouveaux cas diagnostiqués dans les mêmes villages dans le cadre du PNT.
Résultats: Les quatre ONG internationales ont toutes dispensé des soins antituberculeux communautaires dans des contextes difficiles : migrants, zones post-conflit, populations urbaines défavorisées et autres groupes vulnérables. Deux d'entre elles ont recruté des volontaires parmi les agents sanitaires de proximité bénévoles ou les structures de santé existantes, une parmi les autorités des communautés et une autre impliquait directement les sujets infectés ou malades de la tuberculose. Deux ONGI ont indemnisé les volontaires en fonction des résultats et deux autres ont proposé des

incitations financières et en nature. Toutes ont fait appel aux laboratoires du PNT pour le diagnostic et les médicaments mais ont apporté un soutien direct au traitement et assuré son suivi.

Au total, 21 995 cas présumés de tuberculose ont été adressés aux centres de soins pour diagnostic, 7383 (34 %) nouveaux cas ont été diagnostiqués et presque tous (98 %) ont été traités avec succès. Les quatre ONG internationales ont contribué, en moyenne, à la détection de 36 % (7383 sur 20 663) des nouveaux cas de tuberculose dans leurs agglomérations respectives (extrêmes : 15 et 52 %).

Conclusions: Les programmes communautaires de lutte contre la tuberculose des ONG internationales ont réussi à détecter les cas de tuberculose dans les populations isolées et vulnérables. Cette action est indispensable pour réaliser les objectifs de la stratégie de l'OMS pour mettre fin à la tuberculose. Il y aurait lieu d'explorer des stratégies pour pérenniser les programmes, en obtenant notamment un engagement à plus long terme des ONGI.

Translated from English version into French by Suzanne Assenat, through

Организация международными неправительственными организациями лечения туберкулеза на местном уровне для населения труднодоступных районов Мьянмы, 2013–2014

Ко Ту Соэ, Сау Сау, Йохан ван Гринсвен, Шуйсен Джоу, Лей Винь, Паланивель Чиннакали, Сафье Ша, Мио Мио Мон, Си Ту Аун

АННОТАЦИЯ

Общие сведения: Национальные противотуберкулезные программы все чаще привлекают к сотрудничеству международные неправительственные организации (МНГО), в особенности в случаях необходимости организации лечения туберкулеза в сложных условиях, когда требуется участие в общественной деятельности. Однако в Мьянме сведения о том, каким образом организованы данные местные программы МНГО и насколько они эффективны, крайне ограничены. Данное исследование описывает четыре стратегии МНГО по предоставлению лечения туберкулеза на местном уровне для населения труднодоступных районов Мьянмы и дает оценку их вкладу в выявление случаев заболевания туберкулезом.

Методы: В 2013-2014 гг. было проведено описательное исследование, основанное на данных о программах четырех МНГО и Национальной противотуберкулезной программы. По каждой МНГО были выявлены такие сведения, как подход и ключевые виды деятельности, количество предварительно выявленных случаев заболевания туберкулезом и больных, прошедших обследование, а также количество пациентов с подтвержденным диагнозом и результаты их лечения. Вклад МНГО в постановку диагноза туберкулеза на подответственных им территориях был определен путем расчета количественного соотношения диагностированных МНГО новых случаев заболевания туберкулезом к общему числу выявленных случаев заболевания туберкулезом на тех же территориях.

Результаты: Все четыре МНГО внедрили лечение туберкулеза на местном уровне в сложных условиях, ориентируясь на мигрантов, бывшие зоны военных конфликтов, городскую бедноту и другие уязвимые категории населения. Две МНГО наняли волонтеров из местного населения из числа существующих местных

волонтеров-медиков или через местные структуры здравоохранения, одна через действующую местную администрацию и еще одна напрямую привлекла к волонтерской деятельности зараженных туберкулезом больных. Две МНГО оплачивали деятельность волонтеров по результатам деятельности, а две другие использовали системы разового стимулирования или вознаграждения в натуральной форме. Все четыре организации для постановки диагноза и обеспечения противотуберкулезными лекарствами прибегали к помощи лабораторий Национальной противотуберкулезной программы, но при этом напрямую обеспечивали наблюдение больных во время и после прохождения лечения.

В целом, было предварительно выявлено и направлено на обследование 21 995 случаев заболевания туберкулезом. Из них туберкулез был диагностирован в 7383 (34%) случаев и почти все (98%) прошли лечение успешно. Четыре МНГО способствовали выявлению, в среднем, 36% (7383/20663) из общего числа поставленных новых диагнозов туберкулеза на подотчетных им территориях (диапазон: 15-52%).

Выводы: Обеспечиваемая МНГО программа лечения туберкулеза на местном уровне успешно решает вопросы диагностики туберкулеза для уязвимых категорий населения и населения труднодоступных районов. Данные результаты имеют критически важное значение для достижения целей противотуберкулезной стратегии Всемирной организации здравоохранения. Необходимо дополнительно исследовать стратегии, способные обеспечить устойчивость подобных программ, в том числе необходимость в более долгосрочном участии МНГО,

Translated from English version into Russian by Nadia Miretskaya, through

TRANSLATORS
WITHOUT BORDERS

Disposición de las organizaciones internacionales no gubernamentales sobre el tratamiento comunitario de la tuberculosis en poblaciones de difícil acceso de Myanmar, 2013-2014

Kyaw Thu Soe, Saw Saw, Johan van Griensven, Shuisen Zhou, Lei Win, Palanivel Chinnakali, Safieh Shah,Myo Myo Mon, Si Thu Aung

EXTRACTO

Contexto: Cada vez son más los programas nacionales de tuberculosis (TB) que buscan la colaboración de las organizaciones internacionales no gubernamentales (INGOs), especialmente con el fin de proporcionar un tratamiento contra la TB en entornos complicados en los que pueda precisarse la participación comunitaria. Sin embargo, en Myanmar, existen muy pocos datos tanto acerca de la organización, como de la eficacia de estos programas comunitarios de las INGO. En el presente estudio describimos cuatro estrategias de las INGO para proporcionar tratamiento comunitario contra la TB en poblaciones de dificil acceso de Myanmar y evaluamos su contribución a la detección de casos de TB.

Métodos: Llevamos a cabo un estudio descriptivo empleando datos procedentes de los programas de cuatro INGOs así como del Programa Nacional de TB (NTP) de 2013–2014. De cada INGO, extrajimos información acerca de sus actividades clave y de enfoque, sobre el presunto número de casos de TB mencionado y de las pruebas de TB que se estaban realizando, así como del número de pacientes a los que se les diagnosticó TB y de los resultados de sus tratamientos. La contribución de las INGOs al diagnóstico de la TB en cada una de sus poblaciones seleccionadas se

calculó como proporción de nuevos casos de TB diagnosticados por las INGOs en relación con el total de nuevos casos de TB diagnosticados por el NTP en las mismas poblaciones.

Resultados: Las cuatro INGOs aplicaron tratamientos comunitarios contra la TB en situaciones desafiantes, que tenían inmigrantes como objetivo, en áreas donde se acababan de desarrollar conflictos, entre los pobres urbanos y con otros tantos grupos vulnerables. Dos de ellas reclutó a voluntarios comunitarios a través de voluntarios de la salud comunitarios ya existentes o de estructuras sanitarias, otra lo hizo mediante liderazgos comunitarios existentes y la última implicó directamente a individuos infectados de o afectados por la TB. Dos de las INGOs compensó a los voluntarios financiándolos según su rendimiento y otras dos proporcionaron iniciativas financieras y en especie. Todas contaron con los laboratorios del NTP para sus diagnósticos y con los medicamentos de este plan contra la TB, pero proporcionaron ayuda directa en el tratamiento por observación, así como seguimiento en los tratamientos.

Se mencionó un total de 21.995 presuntos casos de TB para su diagnóstico, de los que 7.383 (un 34%) fueron nuevos casos diagnosticados de TB y casi todos (el 98%) fueron tratados con éxito. Las cuatro INGOs contribuyeron a la detección de una media del 36% (7.383/20.663) del total de nuevos casos de TB en sus respectivas poblaciones (rango: 15–52%).

Conclusiones: El tratamiento comunitario de TB apoyado por las INGOs logró con éxito la detección de casos de TB en poblaciones vulnerables y de difícil acceso. Esto es vital para alcanzar las metas de la estrategia contra la TB de la Organización Mundial de la Salud. Deberían estudiarse aquellas estrategias que aseguren la sostenibilidad de los programas, incluida la necesidad de compromiso a largo plazo de las INGOs.

Translated from English version into Spanish by Estartita, through

TRANSLATORS
WITHOUT BORDERS
